# Individuelle Beckenteilersatz-Implantate: 3-D-Planung und Versorgungskonzepte

**DOI:** 10.1007/s00064-023-00826-6

**Published:** 2023-09-19

**Authors:** Martin Wessling, Max Jaenisch, Yannik Hanusrichter, Dieter Christian Wirtz, Carsten Gebert, Thomas Martin Randau

**Affiliations:** 1 Tumororthopädie und Revisionschirurgie, Orthopädische Klinik Volmarstein, Lothar-Gau-Str. 11, 58300 Wetter, Deutschland; 2 Zentrum für muskuloskelettale Chirurgie, Universitätsmedizin Essen, Hufelandstr. 55, 45147 Essen, Deutschland; 3 Klinik und Poliklinik für Orthopädie und Unfallchirurgie, Universitätsklinikum Bonn, Venusberg-Campus 1, 53125 Bonn, Deutschland; 4 Klinik für Allgemeine Orthopädie und Tumororthopädie, Universitätsklinikum Münster, Albert-Schweitzer-Campus 1, 48149 Münster, Deutschland; 5 Klinik für Orthopädie, Spezielle Orthopädische Chirurgie und Sportorthopädie, Krankenhaus der Augustinerinnen, Jakobstr. 27-31, 50678 Köln, Deutschland

**Keywords:** Azetabulum, Knochendefekt, Revisionsendoprothetik, Sonderinstrumente PSI, 3D-Druck, Acetabulum, Bone defect, Revision arthroplasty, Patient-specific instruments PSI, 3D printing

## Abstract

Die Planung und Implantation eines individuellen Beckenteilersatzes stellt hohe Anforderungen sowohl an den Operateur als auch an das gesamte Team (Ingenieur, Assistenten, Operationsteam). Durch die sorgfältige präoperative Planung sowie die stringente intraoperative Durchführung stellt der individuelle Beckenteilersatz ein aufwendiges, aber zuverlässig anwendbares Verfahren zur Defektrekonstruktion auch hochkomplexer azetabulärer Knochendefekte dar, auch nach multiplen Voroperationen.

**Zusatzmaterial online:**

Zusätzliche Informationen sind in der Online-Version dieses Artikels (10.1007/s00064-023-00826-6) enthalten.

## Abstract (en)

The planning and implantation of a customized partial pelvis replacement places high demands on both the surgeon and the entire team (engineer, assistants, surgical team). Thanks to careful preoperative planning and meticulous perioperative execution, customized partial pelvic replacement represents a complex but reliable procedure for defect reconstruction even with highly complex acetabular bone defects or after multiple previous surgeries.

## Vorbemerkungen

Die Anzahl der Revisionseingriffe nach Implantation einer Hüftgelenks-Totalendoprothese (Hüft-TEP) nimmt stetig zu. Das deutsche Endoprothesenregister weist im Operationsjahr 2021 insgesamt 17.700 Folgeeingriffe an der Hüfte aus, die Tendenz zu den Vorjahren ist klar steigend [9]. Primäreingriffe werden großzügiger indiziert [4], und durch die höhere Lebenserwartung und das ebenfalls steigende Aktivitätslevel der Patient*Innen entsteht ein steigender Bedarf an Revisionsoptionen, die ein Mindestmaß an Mobilität erhalten. In komplexen Fällen, insbesondere bei Beckendefekten der „Acetabular Defect Classification“ (ADC) IIIC und IV nach Wirtz [6], ist ein biomechanisch stabiles und medizinisch-funktionell sinnvolles Ergebnis herausfordernd. Die Rekonstruktion kann mit verschiedenen modularen Pfannensystemen durchgeführt werden und stellt sich oft als äußerst komplex und aufwendig dar. Als moderne, technische Möglichkeit stehen seit einigen Jahren die 3‑D-Planung und additive Fertigung (3D-Druck) von individuellen Beckenteilersatz-Implantaten als elegante und einfache Lösungen zur Verfügung [3]. Die Durchführung der präoperativen Planung und verschiedene Konzepte dieser Versorgungsmöglichkeit in der Revisionsendoprothetik werden in diesem Artikel dargestellt.

## Operationsprinzip und -ziel

Das Operationsziel ist die bestmögliche Primärstabilität durch metallische Auffüllung eines Beckendefektes in der Hauptbelastungszone, sichere Kontaktflächen zum Patientenknochen und stabile Verankerung mittels Schrauben oder Zapfen sowie eine gute und langfristige Sekundärstabilität über die knöcherne Integration des individuellen Beckenteilersatzes (BTE) durch raue Implantatoberflächen am Implantatkörper oder an den verwendeten Laschen und Zapfen. Wenn möglich, sollte der notwendige Weichteilschaden minimiert werden.

Das Planungsprinzip und die Fixation orientieren sich hierbei an dem individuellen Defekt, der Erfahrung des Operateurs und den weiteren patientenindividuellen Gegebenheiten. Aufgrund dieser Faktoren ist jeder Beckenteilersatz individuell an die spezifische Situation adaptiert und muss isoliert betrachtet werden, dennoch gibt es allgemeingültige Überlegungen und Prinzipien in der Planung, denen stets besondere Aufmerksamkeit gewidmet werden sollte.

## Vorteile

- Möglichkeit der primärstabilen Verankerung insbesondere bei großen azetabulären Defekten in der Hauptbelastungszone
- Bestmögliche Rekonstruktion von azetabulärem Offset und des Drehzentrums (Center-of-Rotation [COR])
- Größtmöglicher Pfannendurchmesser, zur möglichen Versorgung mittels tripolaren Systems
- Verkürzung von Operationszeiten und Erhöhung der Sicherheit durch Sonderinstrumente („patient-specific instruments“ [PSI]), Bohrschablonen und individualisierte Operationstechnik
- Individuelle, defektadaptierte Auswahl der Fixationsmöglichkeiten

## Nachteile

- In der Regel zweizeitiges Vorgehen notwendig, auch in aseptischen Fällen
- Lange Wartezeit von Indikation bis Implantatbereitstellung (ca. 6 bis 8 Wochen)
- Relativ hohe Implantatkosten, die nur mit entsprechendem Zusatzentgelt (ZE) vergütet werden können
- Notwendige Lernkurve, sowohl in der präoperativen Planung als auch für die Operation selbst
- Fehlende Rückzugsmöglichkeit bei unvorhergesehenem Operationsverlauf

## Indikationen

Die Indikation zur Versorgung mittels eines individuellen Beckenteilersatzes ist häufig keine eindeutige Entscheidung und konkurriert in der Regel mit der Verwendung von modularen Revisionspfannen. Die etablierte Einteilung der Beckendefekte nach Paprosky [5] gewährt keine klare Entscheidungshilfe zur Auswahl eines BTE. Die neue „Acetabular Defect Classification“ (ADC) differenziert insbesondere bei großen kranialen (ADC IIIa) und kraniodorsalen Defekten (ADC IIIb und IIIc) sowie Beckendiskontinuitäten (ADC IV) eindeutiger und bietet eine Empfehlung zum therapeutischem Vorgehen [2].

Zu den typischen Kriterien für einen BTE gehören:

- große kraniale Defekte mit Destruktion des kraniolateralen Erkers („Steilwand-Defekt“),
- Defekte mit destruierter oder retrahierter vorderer und hinterer Acetabulum-Wand und weitem AP-Durchmesser, welcher den der verfügbaren Pfannen übersteigt (in der Regel 72 mm),
- Verlust von Knochensubstanz in der üblichen, zur Verankerung genutzten Region (zentraler Dom im Os ilium, hinterer Pfeiler),
- Ilium-Defekte, welche die Auflage von Laschen oder Stützpfeilerplatten („buttress plates“) unmöglich machen,
- Beckendiskontinuitäten, die eine primärstabile Verankerung einer Pfanne unmöglich machen,
- insuffiziente Rekonstruktion von COR und Offset mit Standardimplantaten (oft Kranialisierung und Lateralisierung des COR durch die Verwendung übergroßer hemisphärischer Pfannen) [7],
- fehlende Primärstabilität eines komplexen modularen Konstrukts.

Die Entscheidung zum BTE wird aber letztlich immer von der Erfahrung und den Vorlieben des Operateurs beeinflusst.

Zu den Kontraindikationen gehören:

- einzeitiger septischer Wechsel (da Verankerung in der Regel zementfrei, damit fehlende lokale Antibiose als wesentlicher Pfeiler der Infekttherapie),
- persistierender Infekt im Operationsgebiet beim zweizeitigen Wechsel,
- Unmöglichkeit des zweizeitigen Vorgehens (z. B. aufgrund von Narkoserisiken oder anderer Nebendiagnosen),
- fehlende Compliance oder Bereitschaft des Patienten zum 6‑ bis 8‑wöchigen Intervall zwischen Aus- und Wiedereinbau,
- dauerhaft zu erwartende Immobilität des Patienten.

## Patientenaufklärung

Die Patientenaufklärung erfolgt anhand von entsprechenden Aufklärungsbögen „Hüft-TEP-Wechsel“ und muss einigen besonderen Aspekten Rechnung tragen:

- Aufklärung über den Einsatz eines nicht-CE-zertifizierten Sonderimplantates im Sinne des „Compassionate Care“ trotz der prinzipiellen Verfügbarkeit zugelassener Standardimplantate,
- Aufklärung über Modifikation des operativen Vorgehens nach intraoperativem Befund,
- schlechtestenfalls Unmöglichkeit des erfolgreichen Operationsabschlusses,
- zweizeitiges Vorgehen auch im aseptischen Fall zur besseren und sichereren Planung des Implantates,
- prothesenfreies Intervall, ggf. mit Spacer oder Liner, dadurch nötige Entlastung der Extremität und ggf. resultierende Immobilisierung in dieser Zeit mit allen möglichen Komplikationen (Thrombose, Embolie, Dekubitus, Lungen- und Harnwegsinfekte, Pflegebedürftigkeit …),
- Einwilligung des Patienten zur Weitergabe seiner Krankheitsdaten und Bildmaterial an die fertigende Firma (s. Anhang 1),
- Mix-and-Match bei Verwendung von z. B. tripolaren Pfannenkomponenten, falls der Hersteller diese nicht bereitstellt.

## Operationsvorbereitungen

- Präoperative Diagnostik – Ausschluss einer periprothetischen Infektion
  - Punktion des Gelenkes mit Zellzählung und Differenzierung
  - Punktat zur mikrobiologischen Diagnostik mit Kultur und molekularbiologischer Diagnostik (Multiplex- oder 16S-Polymerase-Kettenreaktion [PCR])
  - Mikroskopie des Punktates
- Bestimmung von Infektparametern im Serum, ggf. Fokussuche und -sanierung
  - Harnwege
  - Pulmonale Infekte
  - Zahnstatus
- Patient Blood Management (PBM) zur Optimierung des präoperativen Status bei planbaren Eingriffen, spätestens vor der Reimplantation
- Werden Implantate belassen (z. B. der Prothesenschaft), so müssen alle notwendigen Teile zur Revision beim Wiedereinbau verfügbar sein (passende Steckköpfe, Adapterhülsen, bei modularen Schäften alternative Halsteile, Verlängerungshülsen etc.)
- Sozialdienst zur Regelung der Versorgung im prothesenfreien Intervall
- Bei einliegenden Metall-Metall-Implantaten oder Konus-Steck-Verbindungen ggf. Kobalt- und Chromlevel im Serum des Patienten zum Ausschluss einer systemischer Metallose
- Allergietestung zum Ausschluss von Unverträglichkeiten gegen verwendete Materialien
- Regelung der Kostenübernahme mit den Kostenträgern, sofern kein ZE verhandelt wurde
- Kontaktaufnahme mit einem geeigneten Hersteller zur gemeinsamen Planung und Herstellung des Implantates

## Anästhesie und Lagerung

- Intubationsnarkose mit zumindest zeitweiser Muskelrelaxation
- Ausreichend Blutprodukte und ggf. Cell-Saver, wenn möglich
- Epiduralkatheter zur intra- und postoperativen Schmerztherapie
- Durchleuchtbarer Operationstisch (z. B. Karbontisch)
- Lagerung für den jeweiligen Hüftzugang: Bewährt hat sich die Seitenlage auf einer Vakuummatratze, die ein posteriores, laterales oder anterolaterales Zugehen erlaubt
- Abdeckung gemäß dem gewählten Zugang. Die Abdeckung sollte einen ilioinguinalen Zugang zum Komplikationsmanagement erlauben

## Operationstechnik

**Figure 1:**
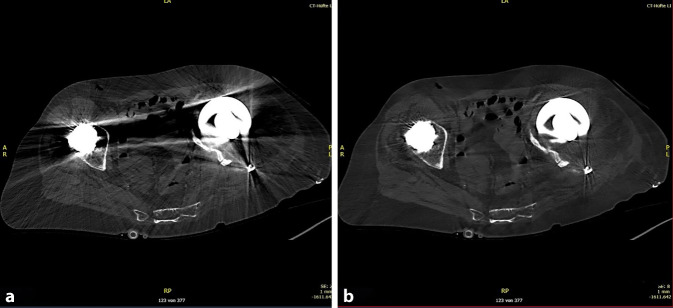

**Figure 2:**
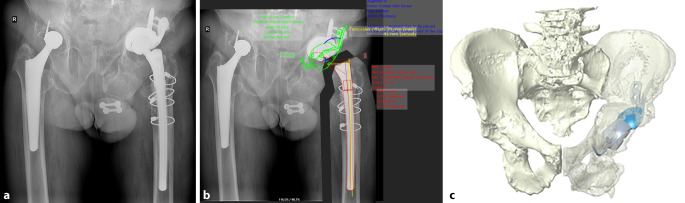

**Figure 3:**
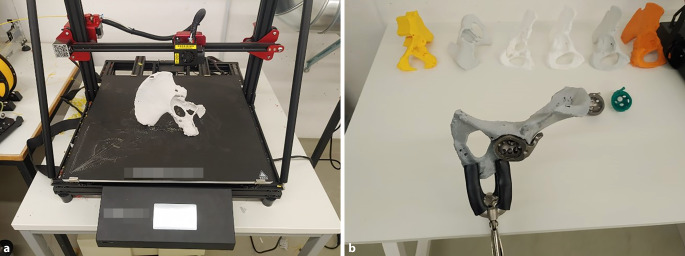

(Abb. 1, 2, 3, 4 und 5)

**Figure 4:**
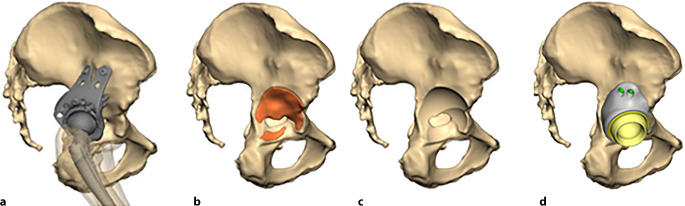

**Figure 5:**
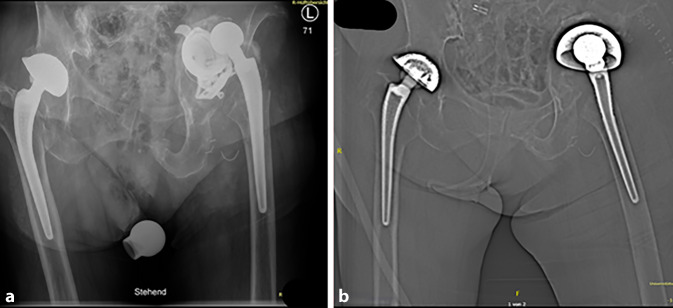

Soll ein Fall zum BTE evaluiert oder geplant werden, so sind im ersten Schritt einem gewählten Hersteller die Bilddateien zusammen mit allen relevanten klinischen Angaben (Krankheitsverlauf, einliegendes Implantat, Größe, Gewicht und Aktivitätslevel des Patienten) zur Verfügung zu stellen. Welcher Hersteller gewählt wird, ist eine subjektive Entscheidung und hängt neben z. B. noch im Patienten verbliebenen Implantatkomponenten, Lieferverfügbarkeit im gewünschten zeitlichen Rahmen v. a. von der Erfahrung und Bewertung der einzelnen Hersteller ab. Wir empfehlen, die ersten Implantate von stets demselben Hersteller planen zu lassen und zu beziehen, um eine gemeinsame Lernkurve zu ermöglichen. Bei hoher Auftragsdichte oder um die eigene Erfahrung zu verbreitern, können später andere Lieferanten einbezogen werden.

Fast alle Hersteller bieten entsprechende Upload-Portale und Online-Dashboards zur Verwaltung der Fälle und zur Verfolgung des Planungsprozesses an. Das Einverständnis des Patienten ist aus Datenschutzgründen vorher einzuholen und schriftlich zu dokumentieren (s. Anlage 1, „Einverständnis zur Datenübermittlung Individualimplantat“). Zu Beginn einer Planung sollte mit dem Hersteller geklärt sein, wie lange die erwartete Fertigung dauert, wie die Daten übermittelt werden sollen und wie der eigentliche Planungsprozess und die Kommunikation mit dem Ingenieur erfolgen sowie ggf. der erwartete Preisrahmen für das Implantat und mögliche Modelle und Schablonen.

Einen Patientenfall mehreren Herstellern simultan zur Planung zu übersenden ermöglicht zwar Vergleiche der Planungen und Konstruktionen untereinander und letztlich auch einen Preisvergleich der Hersteller gegeneinander, verursacht aber andererseits erheblichen Zeit- und Arbeitsaufwand aufseiten der Hersteller. Wird diese Möglichkeit überreizt, so darf es nicht verwundern, wenn seitens des Herstellers irgendwann schon für die bloße Planung ein Kostenvoranschlag gesendet wird und die interne Priorisierung der übersendeten Fälle sinkt.

(Abb. 6, 7, 8, 9, 10, 11, 12, 13, 14, 15, 16, 17, 18, 19, 20, 21, 22, 23, 24, 25, 26, 27 und 28)

**Figure 6:**
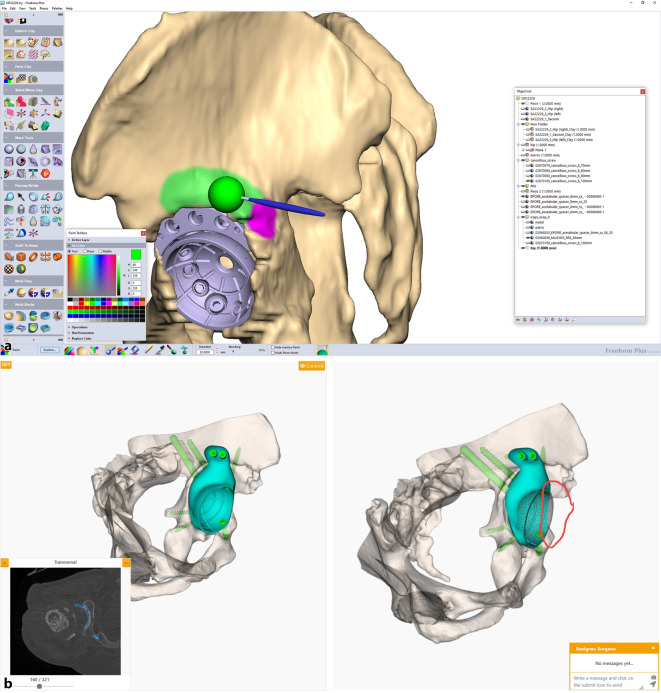

**Figure 7:**
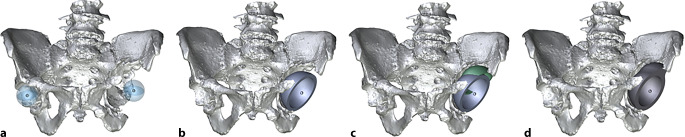

**Figure 8:**
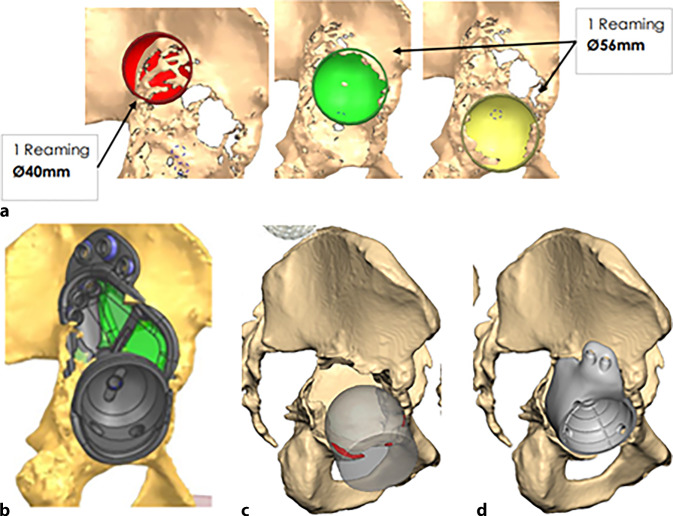

**Figure 9:**
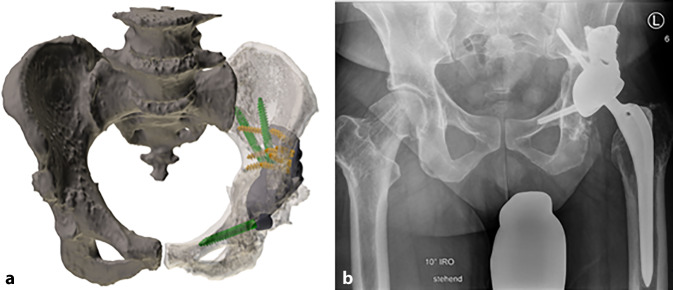

**Figure 10:**
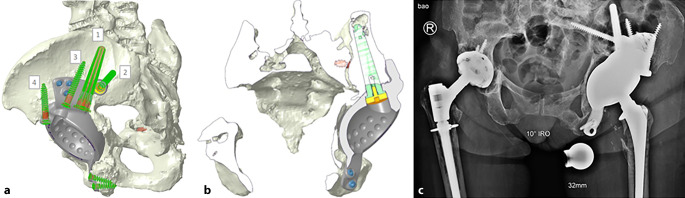

**Figure 11:**
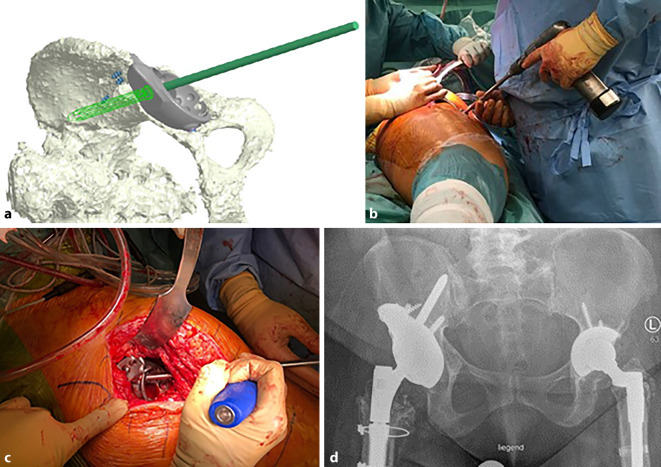

**Figure 12:**
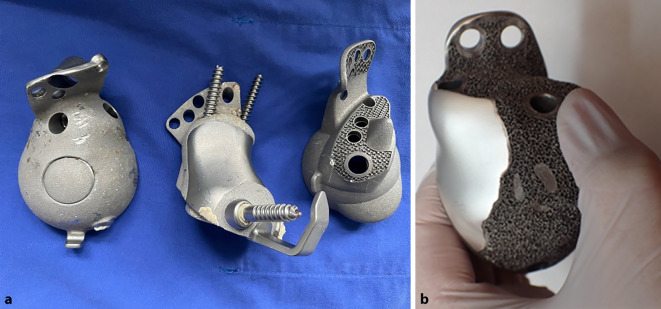

**Figure 13:**
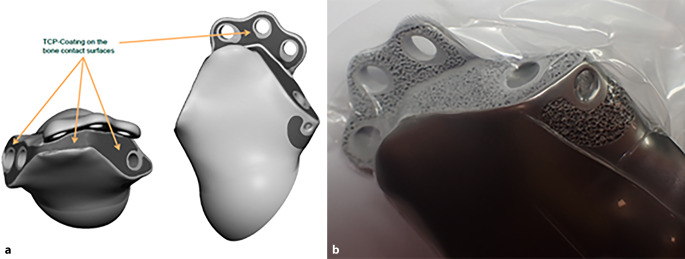

**Figure 14:**
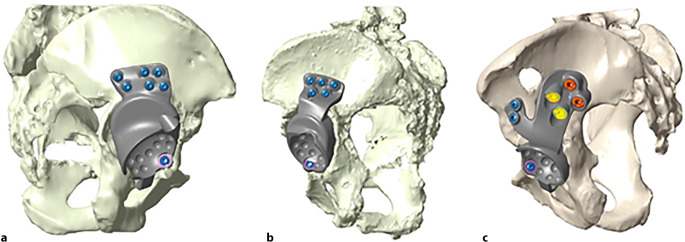

**Figure 15:**
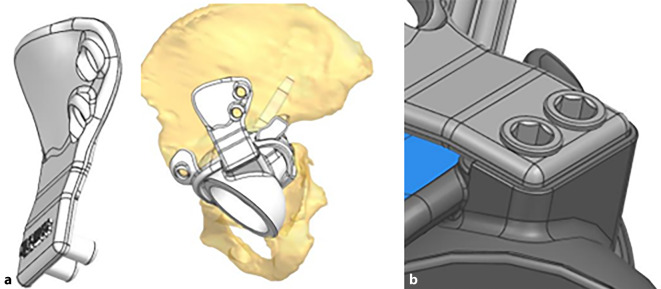

**Figure 16:**
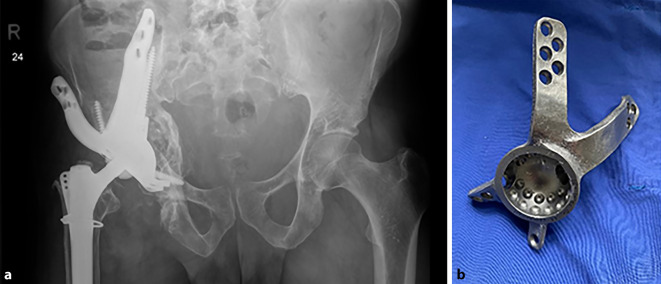

**Figure 17:**
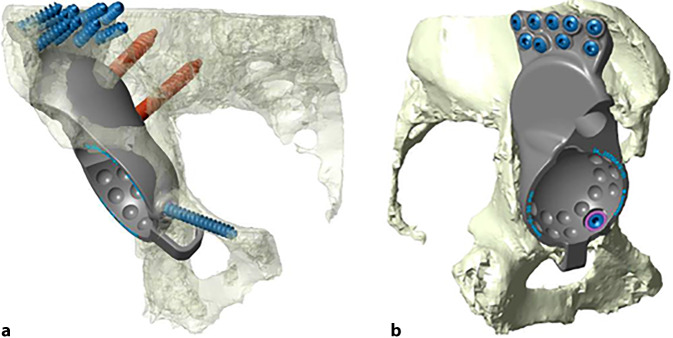

**Figure 18:**
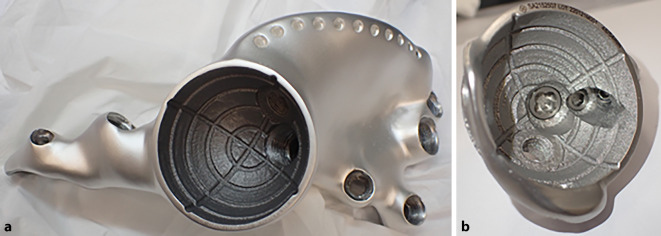

**Figure 19:**
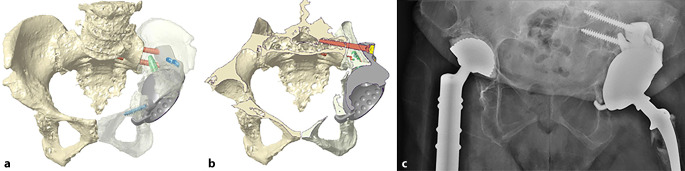

**Figure 20:**
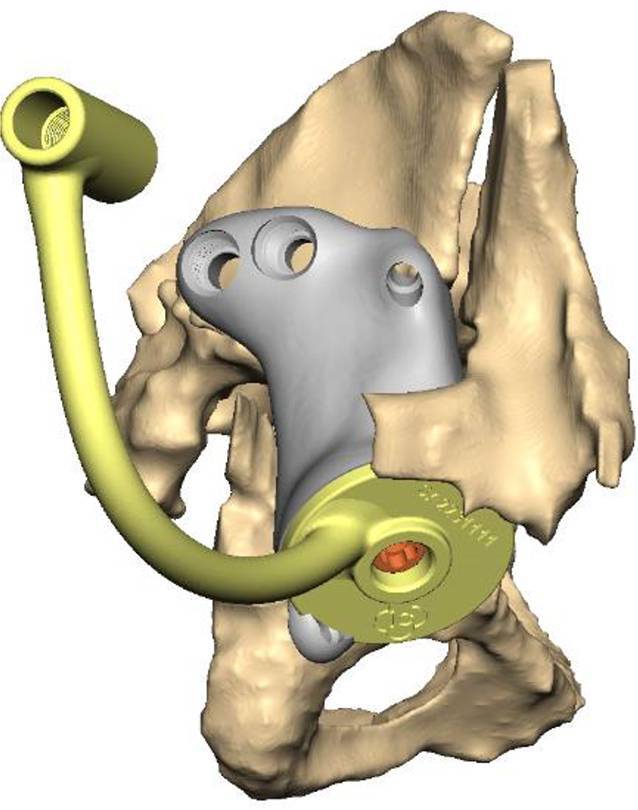

**Figure 21:**
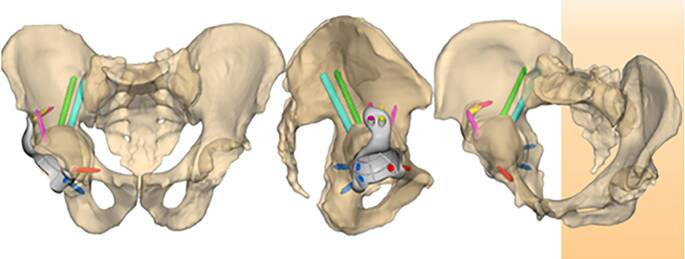

**Figure 22:**
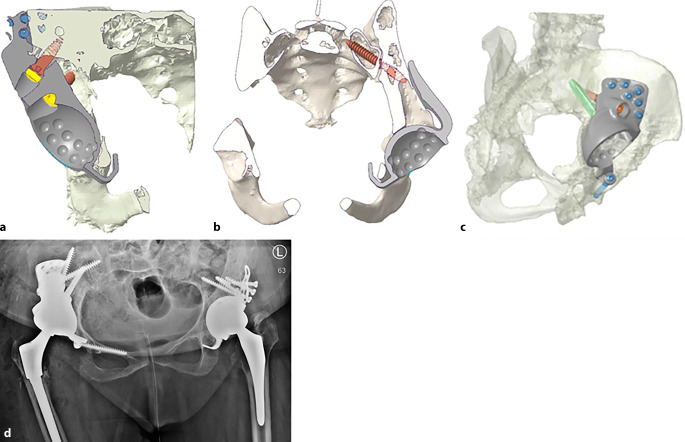

**Figure 23:**
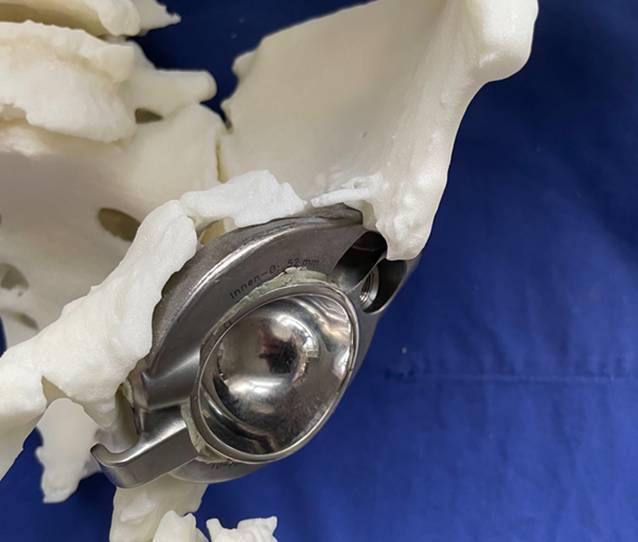

**Figure 24:**
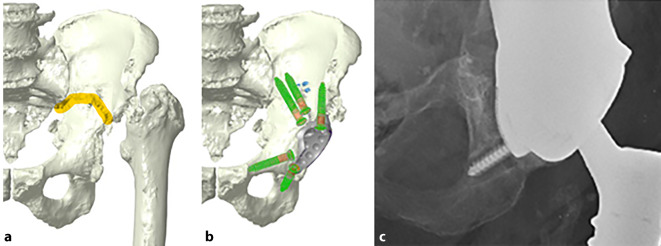

**Figure 25:**
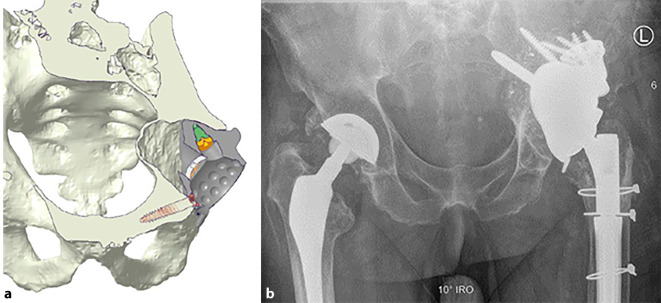

**Figure 26:**
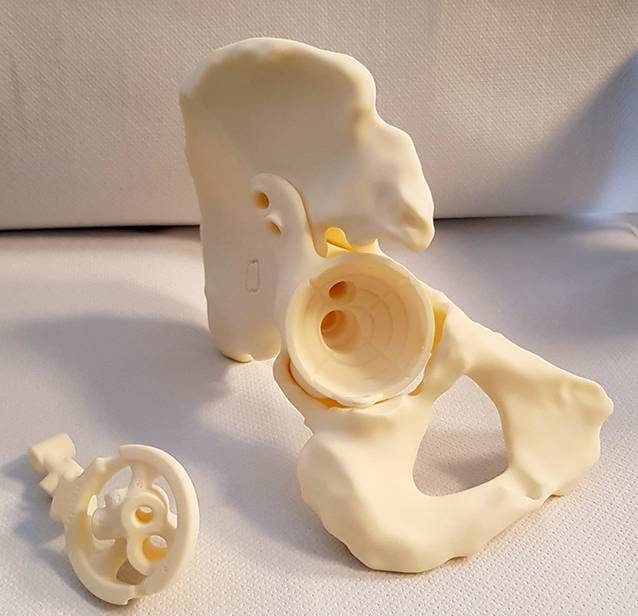

**Figure 27:**
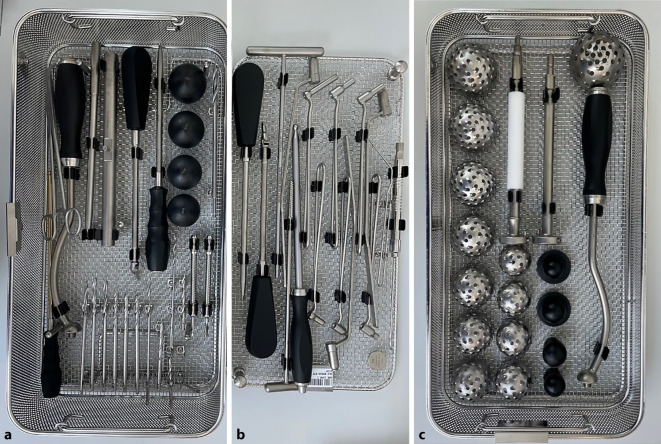

**Figure 28:**
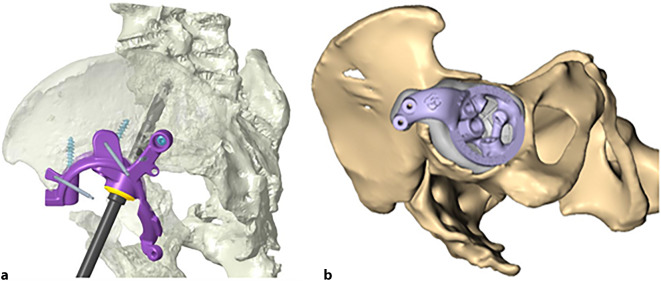

## Zugangswege

Der Zugangsweg sollte, wenn möglich, dem bereits vorbestehenden entsprechen, aber auch eine gute Exposition der notwendigen knöchernen Grenzen ermöglichen, die für die Implantation dargestellt werden müssen. In aller Regel kann ein BTE über einen anterolateralen, lateralen oder posterioren Hüftzugang implantiert werden. Die Bohrrichtung der Schrauben sollte dem Zugang entsprechend gewählt werden.

## Intraoperative Bildgebung

Die individuelle Passform des Implantates, kombiniert mit entsprechenden Modellen und Bohrschablonen, macht die Implantat- und Schraubenpositionierung im Vergleich zu Standardimplantaten eher leichter. Trotzdem sollte für die korrekte Positionierung des Implantates und der Schrauben die Möglichkeit zur intraoperativen Bildgebung obligat vorhanden sein (C-Bogen, Bildverstärker). Bei Lagerung und Abdeckung muss darauf geachtet werden, dass alle gängigen Ebenen (Becken anteroposterior, Inlet- und Outlet-Aufnahme, Hüfte axial nach Lauenstein sowie Ala- und Obturator-Zielaufnahme) eingestellt werden können. Ein 3‑D-Bildverstärker kann eingesetzt werden, sofern dieser vorhanden ist, ist aber nicht zwingend notwendig – alle hier vorgestellten Fälle aus beiden Zentren wurden ohne 3‑D-Bildverstärker operiert.

## Postoperative Behandlung

Die postoperative Behandlung unterscheidet sich nicht wesentlich von der Behandlung einer normalen Hüftpfannenrevisionsoperation:

- nativradiologische Kontrolle der Implantatlage,
- großzügige Indikation zum postoperativen CT zur Kontrolle der Schraubenlage; ein Abgleich der Bilder mit der Planung verbessert zudem die zukünftige Planung,
- Vollbelastung, wenn vertretbar,
- Vermeiden von Extrembelastungen für 3 Monate,
- Restriktion der Hüftflexion auf 60° und der IRO/ARO für 6 Wochen,
- Kontrolle der Mikrobiologie und Pathologiebefunde,
- Kontrolle Röntgen nach 6 bis 12 Wochen,
- Thromboseprophylaxe für 4 bis 6 Wochen.

Erfolgt die Reimplantation des Sonderimplantates im Rahmen der zweiten Operation eines zweizeitigen, septischen Wechsels, so ist hinsichtlich der antibiotischen weiteren Therapie postoperativ eine antibiogrammgerechte und biofilmwirksame Antibiose fortzusetzen; Bei aseptischen Revisionsgründen gibt es keine Evidenz hinsichtlich einer längeren antibiotischen Therapie jenseits der perioperativen Prophylaxe für maximal 24 h [8], wenn intraoperativ kein Hinweis auf einen Infekt besteht.

## Fehler, Gefahren, Komplikationen

Der gesamte Planungs- und Fertigungsprozess eines individuellen Beckenteilersatzes stellt aufgrund der potenziellen Unsicherheiten und möglicher Fehler nur eine Näherung an die reellen Gegebenheiten dar. Diese beginnt mit der Erstellung eines CT-Abbildes vom Becken, dem Sequenzieren eines knöchernen 3‑D-Modells aus diesem Planungs-CT und dem nachfolgenden digitalen Planen eines BTE mit anschließender Fertigung und dann Implantation mit dem geplanten Zugangsweg unter Schonung von Muskulatur, Gefäßen und Nerven und der individuellen Physiognomie des Patienten. Fehler werden vermieden durch ein ausreichend erfahrenes Team in der Planung und Durchführung der Operation; ggf. sollte, insbesondere in den ersten Fällen, von extern ein erfahrener Kollege oder eine erfahrene Kollegin hinzugezogen werden.

Liegen zwischen Planungs-CT und Beginn der Fertigung oder zwischen Fertigung und Reimplantation mehr als ein paar Wochen, so kann sich der knöcherne Defekt signifikant verändert haben. Ein neues CT vor Reimplantation kann zum Abgleich mit dem bereits geplanten oder sogar schon gefertigten Implantat dienen und zeigen, an welchen Stellen nachpräpariert werden muss oder wo und inwieweit die Passgenauigkeit kompromittiert ist.

Eine Beurteilung der Knochenqualität ist im CT nicht immer sicher möglich, besonders wenn verbliebenes Implantatmaterial zu Artefakten und Überlagerungen führt. Eine Fehleinschätzung hinsichtlich der Knochenqualität kann die primärstabile Verankerung deutlich erschweren. Redundante Verankerungsmöglichkeiten (Lasche, Schraube, Darmbeinzapfen) in verschiedenen Positionen helfen, dieses Problem abzufangen.

Die weite Exposition der Beckenaußenseite birgt die Gefahr von Verletzungen der Gefäße, insbesondere der Aa. gluteae und des N. ischiadicus. Die Erfahrung des Operateurs und die genaue Planung des Implantates in Bezug zu diesen Strukturen helfen hier, Komplikationen zu vermeiden. Ein gefäßchirurgisches Back-up ist trotzdem empfehlenswert.

Das Einbringen langer Schrauben durch das Becken kann auch bei geringer Gradabweichung des Eintrittswinkels zu einer intrapelvinen Schraubenlage führen mit Beschädigung großer Gefäße (A./V. iliaca) und Nerven (Plexus sacralis). Gründliche radiologische Kontrolle in mehreren Ebenen hilft, die Bohrer- und Schraubenlage zu verifizieren (Inlet, Outlet, Ala, Obturator, AP und lateral), der Patient sollte entsprechend auf einen durchleuchtbaren Tisch gelagert sein. Die sterile Abdeckung ist zudem so zu wählen, dass ein Zugang zu diesen Gefäßen, z. B. durch einen LeTournel-Zugang, rasch und jederzeit möglich ist.

Insbesondere bei langem prothesenfreiem Intervall kann die Rekonstruktion der Beinlänge schwierig sein. Insgesamt planen wir im überwiegenden Teil der Fälle mit einer anatomischen Rekonstruktion des Rotationszentrums azetabulär. Wird der Schaft nicht mit gewechselt, so ist mitunter ein umfängliches femorales Release nötig, um nach langer Girdlestone-Situation eine Reposition überhaupt zu ermöglichen. Neben trotzdem verbleibenden Unterschieden in der Beinlänge gehören neurologische Traktionsschäden zu den Risiken dieses Vorgehens.

Weitere typische postoperative Komplikationen sind die Gelenkluxation und die periprothetische Infektion. Das Risiko für Ersteres kann durch die Verwendung tripolarer Pfannen und eine korrekte Pfannenpositionierung sowie ggf. die Verlängerung des femoralen Offsets mit entsprechenden Kopfadaptern minimiert werden. Zur Infektionsprophylaxe sollten alle Maßnahmen ergriffen werden, die auch in der Revisionsendoprothetik Einsatz finden. Zudem sind die Operationsdauer und der Weichteilschaden durch ein erfahrenes Operationsteam auf ein minimal Nötiges zu reduzieren [1].

## Ergebnisse

Im Zuge der retrospektiven Auswertung zweier großer Revisionszentren wurden alle Planungen und Implantationen von BTEs von 2019 bis 2022 analysiert. Von insgesamt 98 geplanten und zur Fertigung freigegebenen Implantaten wurden 95 implantiert, relativ gleich verteilt auf beide Zentren (1: 49 Stck, 2: 46 Stck); Eine Patientin ist vor der Reimplantation verstorben, eine Patientin entschied sich gegen die Reimplantation, der dritte Patient zeigte keine hinreichende Infektkonsolidierung und wurde als Girdlestone-Hüfte belassen.

Das Durchschnittsalter der Patienten betrug 68,4 Jahre (StdAbw ± 13,5; Range: 30–88); 71 % (*n* = 68) der Patienten waren weiblich. In 35 Fällen war der Grund für den Prothesenwechsel ein periprothetischer Infekt, in 49 Fällen eine aseptische Lockerung; in den verbliebenen 11 Fällen lagen andere Gründe vor (Frakturen, Deformitäten etc.). Primäre Knochentumorbehandlungen sind in dem analysierten Kollektiv explizit ausgeschlossen worden, da hier maßgeblich andere Kriterien für die Planung und Operation zum Tragen kommen (Resektionsgrenzen etc.).

Insgesamt wurden BTEs von 5 verschiedenen Herstellern geplant und implantiert (Peter Brehm, implantcast, Lima Promade, AQ-Implants und Link), wobei der überwiegende Anteil der Implantate von den ersten beiden Herstellern stammte. Von diesen wurde ausgewertet, wie viel Zeit zwischen Bereitstellung der Bilder bis zur Implantation verging, im Durchschnitt waren dies 73,2 Tage (StdAbw ± 34,3 Tage, Range: 22 bis 226 Tage). Zahlreiche Faktoren können diesen Wert beeinflussen, jedoch zeigt sich, dass mindestens 3 Wochen (in der Regel ohne Beschichtung und mit unsteriler Lieferung) und durchschnittlich 6 bis 14 Wochen bis zur Reimplantation veranschlagt werden sollten.

Hinsichtlich der Kosten der Implantate wurde keine vollständige Analyse durchgeführt. In unserer Erfahrung ist ein Bruttopreis von 9000 bis 12.000 € für das Implantat zu erwarten. Je nach Hersteller werden Planungsaufwand, Modelle, PSIs, Operationsbegleitung, Leihinstrumente etc. getrennt berechnet oder als Paketpreis in Rechnung gestellt. Die Verhandlung eines entsprechenden krankenhausindividuellen Zusatzentgeltes, z. B. über den OPS-Code 5‑785.4d, ist zur kostendeckenden Erbringung der Leistung absolut empfehlenswert.

**Figure 29:**
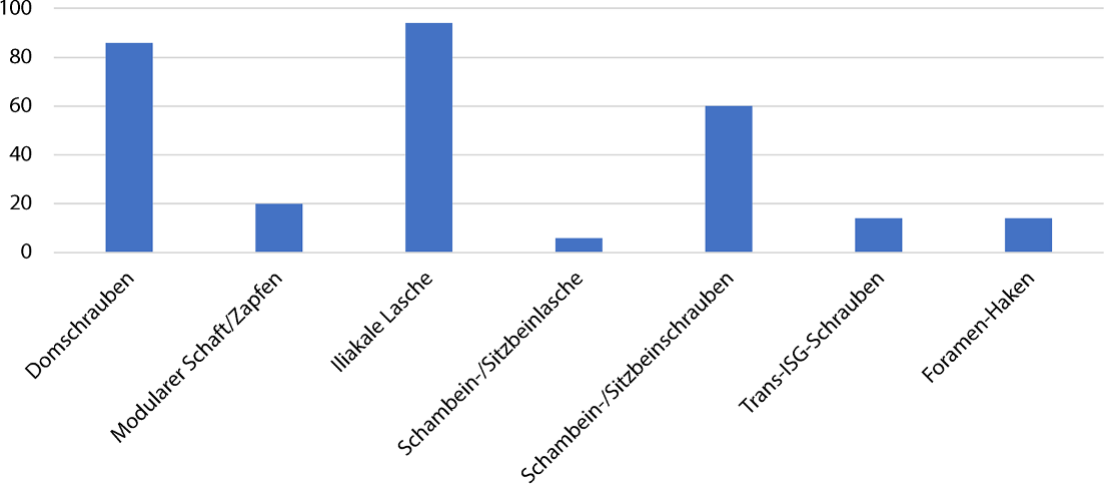

Die Abb. 29 zeigt, welche Methoden der Fixation wie häufig angewendet wurden. Im überwiegenden Teil der Fälle wurden eine iliakale Lasche (*N* = 94) und eine oder mehrere Domschrauben (*N* = 86) eingesetzt; in 20 Fällen kam anstelle oder zusätzlich zur Domschraube ein modularer Zapfen oder Schaft zum Einsatz. Eine zusätzliche Fixierung im Schambein oder im Sitzbein wurde in knapp zwei Drittel der Fälle (*N* = 60) benutzt, hier aber mit deutlicher Präferenz eines der Zentren. Im Gegenzug wurde die zusätzliche Verwendung eines Hakens im Foramen obturatorium (*N* = 14) relativ exklusiv im anderen Zentrum verwendet. Transiliosakrale Schrauben kamen relativ selten zum Einsatz (*N* = 14), ebenso wurde sehr selten auf zusätzliche Laschen auf Sitz- oder Schambein im Sinne eines Triflanges zurückgegriffen (*N* = 6).

Alle Implantate wiesen eine Oberflächenmodifikation der Knochenkontaktflächen auf: In 41 Fällen war das Implantat rau gestrahlt, in den übrigen 54 Fällen kam eine makroraue bzw. trabekuläre Struktur zum Einsatz. TCP- oder HA-Beschichtungen wurden in diesem Kollektiv nicht verwendet.

Die Schnitt-Naht-Zeit für die Implantation betrug im Durchschnitt 240,9 min (StdAbw ± 64,1 min, Range: 113–490 min), und die Patienten waren im Durchschnitt 18 Tage (± 10,2 Tage, Range 6 bis 43 Tage) stationär.

### Supplementary Information




