# Supplementary material for: Customized partial pelvis replacement: three-dimensional planning and management concepts
Source: Oper Orthop Traumatol. 2023 Sep 19;35(5):278–97. [Article in German] doi: 10.1007/s00064-023-00826-6 (PMC10520193; doi:10.1007/s00064-023-00826-6)
Supplement: Supplementary file 1 [file 64_2023_826_MOESM1_ESM.docx]

PID: _________________________________

Geburtsdatum: _________________________________

Sehr geehrte Dame, sehr geehrter Herr,

im Rahmen des geplanten operativen Eingriffes an _____________________________________ ist die Implantation eines Sonderimplantates, bzw. eines individuell gefertigten Implantates (“PMI, Patient-Matched Implant”) geplant. Die Fertigung dieses Implantates erfolgt nicht durch die Klinik selbst, sondern durch einen spezialisierten Hersteller. Zur optimalen Herstellung benötigt die Firma nebst der angefertigten radioloischen Bilder (CT und/oder normale Röntgenbilder) auch klinische Informationen wie - zum Beispiel, aber nicht nur - OP-Berichte, Arztbriefe und Befunde aus der Anamnese, die für den Fall relevant sind. Dieser Vorgang ist in dieser Form gängige Praxis.

Wir bitten Sie daher, dieses Formblatt zur Einverständnis-Erklärung auszufüllen und zu unterschreiben.

**Einwilligungserklärung zur Datenweitergabe**

Ich wurde von meinem Arzt ____________________ daüber informiert, dass die vorhandenen radiologischen Daten an die Firma _____________________________, im Folgenden als “Hersteller” bezeichnet, übergeben werden, und erkläre dazu hiermit mein Einverständnis.

Ich bin ferner damit einverstanden, dass alle relevanten Daten aus der medizinischen Dokumentation (zum Beispiel, aber nicht ausschließlich, Arztbriefe, Operationsberichte, Laborergebnisse, Befunde aus der Anamnese) dem Hersteller übergeben und vom Hersteller und vom KLINIKUM XX zu folgenden Zwecken verwendet werden dürfen: Planung und Herstellung eines Individual-Implantates oder Planung von modularen Implantaten, wissenschaftliche Forschung, Überwachung der Produktsicherheit, wissenschaftliche Dokumentation und Datenerhebung und wissenschaftliche Publikation.

Dokumente, die an den Hersteller weitergeleitet werden, enthalten zwecks Vermeidung von Verwechslungen auch persönliche Informationen, wie Name und Geburstdatum. In der Verwendung ausserhalb der Klinik und des Herstellungsprozesses werden diese Daten pseudonymisiert, so dass ein direkter Rückschluß auf meine Person für Dritte nicht möglich ist.

Ich nehme zur Kenntnis und willige ein, dass die Daten für die vorgenannten Zwecke auch an Gesellschaften außerhalb des europäischen Wirtschaftsraumes übermittelt werden können. Ich wurde darüber informiert und mir ist bewusst, das alle persönlichen Daten streng vertraulich behandelt und auf Grundlage der geltenden nationalen und internationalen Datenschutzgesetze verarbeitet werden.

Abschließend wurde ich darüber informiert und ich bin mir bewusst, dass meine Einwilligung jederzeit mittels schriftlicher Erklärung an das KLINIKUM XX oder direkt an den Hersteller widerrufen werden kann. Im Falle eines Widerrufs ist weder der Hersteller, noch das KLINIKUM XX, haftbar für etwaige vor dem Erhalt der Widerrufserklärung durchgeführte Maßnahmen im Sinne der Datenverarbeitung.

*Ich* *habe die Informationen in diesem Dokument mit meinem Arzt und/oder anderen Vertrauenspersonen durchgesprochen. Es war ausreichend Zeit vorhanden, um die oben aufgeführten Informationen zu lesen, zu verstehen und zu überdenken. Ich habe alle Fragen, die mir in diesem Zusammenhang in den Sinn kamen, gestellt, und diese wurden zu meiner Zufriedenheit beantwortet.*

Unterzeichnet in __________________________________, am _______________________

(Ort der Unterzeichnung der Einwilligungserklärung) (Datum)

Unterschrift des Patienten oder des gesetzlichen Vertreters __________________________________
